# Supplementary material for: Adverse health outcomes in offspring of parents with alcohol-related liver disease: Nationwide Danish cohort study
Source: PLoS Med. 2024 Oct 23;21(10):e1004483. doi: 10.1371/journal.pmed.1004483 (PMC11540217; doi:10.1371/journal.pmed.1004483)
Supplement: S1 Table — (DOCX) [file pmed.1004483.s001.docx]

Supplementary Table S1. Included data sources.

The National Patient Registry contains information on all inpatient contacts since 1977, and since 1995, information on all outpatient contacts. Data include dates of admission and discharge and discharge diagnosis codes. Diagnoses were coded according to the International Classification of Diseases, 8^th^ edition (ICD-8) from 1977 through 1993, and since 1994 according to ICD-10.

The Danish Cause of Death Registry holds data about the cause of death, death date, place of death, manner of death, and autopsy information. The causes of death are recorded as underlying, immediate, or contributory causes and coded according to WHO’s tenth international classification (ICD-10).

The Danish Civil Registration System contains data on date of death and migration to and from Denmark, allowing unambiguous linkage between registries, extraction of population controls, and complete tracking of Danish inhabitants. The Danish Civil Registration System continuously updates data on parent-child relations, dates of birth, death, immigration, and emigration. Nearly all children born after 1960 can be identified in the registry, and some born before 1960 can be identified, too. Biological and adoptive relatives cannot be distinguished, and approximately 1% of a Danish birth cohort is adopted.

The Population Education Register contains Information on the highest completed level of education, derived from type and duration of schooling. About 3% of the population have unknown educational status, either because they are immigrants to Denmark or because their education is not acknowledged by Danish authorities.
