# Supplementary material for: Adverse health outcomes in offspring of parents with alcohol-related liver disease: Nationwide Danish cohort study
Source: PLoS Med. 2024 Oct 23;21(10):e1004483. doi: 10.1371/journal.pmed.1004483 (PMC11540217; doi:10.1371/journal.pmed.1004483)
Supplement: S2 Table — (DOCX) [file pmed.1004483.s002.docx]

Supplementary Table S2. The 10 most frequent hospital contacts in offspring and comparators

| *Alcohol-specific diagnosis* | | | |
| --- | --- | --- | --- |
| *Offspring* | | *Comparators* | |
| ICD-10 | Name | ICD-10 | Name |
| F100 | Mental and behavioural disorders due to use of alcohol : acute intoxication | F100 | Mental and behavioural disorders due to use of alcohol : acute intoxication |
| F102 | Mental and behavioural disorders due to use of alcohol : dependence syndrome | F102 | Mental and behavioural disorders due to use of alcohol : dependence syndrome |
| F101 | Mental and behavioural disorders due to use of alcohol : harmful use | F101 | Mental and behavioural disorders due to use of alcohol : harmful use |
| F103 | Mental and behavioural disorders due to use of alcohol withdrawal state | F103 | Mental and behavioural disorders due to use of alcohol withdrawal state |
| K703 | Alcoholic cirrhosis of liver | T519 | Toxic effect of alcohol |
| T519 | Toxic effect of alcohol | K703 | Alcoholic cirrhosis of liver |
| F104 | Mental and behavioural disorders due to use of alcohol withdrawal state with delirium | T510 | Acute alcohol intoxication |
| T510 | Acute alcohol intoxication | F104 | Mental and behavioural disorders due to use of alcohol withdrawal state with delirium |
| F109 | Mental and behavioural disorders due to use of alcohol : unspecified mental and behavioural disorder | F109 | Mental and behavioural disorders due to use of alcohol : unspecified mental and behavioural disorder |
| K704 | Alcoholic hepatic failure | K704 | Alcoholic hepatic failure |
| *Other abuse diagnosis* | | | |
| *Offspring* | | *Comparators* | |
| ICD-10 | Name | ICD-10 | Name |
| F171 | Mental and behavioural disorders due to use of tobacco: harmful use | F171 | Mental and behavioural disorders due to use of tobacco: harmful use |
| F122 | Mental and behavioural disorders due to use of cannabinoids : dependence syndrome | F122 | Mental and behavioural disorders due to use of cannabinoids : dependence syndrome |
| F192 | Mental and behavioural disorders due to multiple drug use and use of other psychoactive substances : dependence syndrome | F192 | Mental and behavioural disorders due to multiple drug use and use of other psychoactive substances : dependence syndrome |
| F112 | Mental and behavioural disorders due to use of opioids : dependence syndrome | F121 | Mental and behavioural disorders due to use of cannabinoids : harmful use |
| F121 | Mental and behavioural disorders due to use of cannabinoids : harmful use | F112 | Mental and behavioural disorders due to use of opioids : dependence syndrome |
| F191 | Mental and behavioural disorders due to multiple drug use and use of other psychoactive substances : harmful use | F172 | Mental and behavioural disorders due to use of tobacco : dependence syndrome |
| F172 | Mental and behavioural disorders due to use of tobacco : dependence syndrome | F191 | Mental and behavioural disorders due to multiple drug use and use of other psychoactive substances : harmful use |
| F195 | Mental and behavioural disorders due to multiple drug use and use of other psychoactive substances : psychotic disorder | F132 | Mental and behavioural disorders due to use of tobacco : amnesic syndrome |
| F132 | Mental and behavioural disorders due to use of tobacco : amnesic syndrome | F195 | Mental and behavioural disorders due to multiple drug use and use of other psychoactive substances : psychotic disorder |
| F152 | Mental and behavioural disorders due to use of other stimulants, including caffeine : dependence syndrome | F125 | Mental and behavioural disorders due to use of cannabinoids : psychotic disorder |
| *Psychiatric disease diagnosis* | | | |
| *Offspring* | | *Comparators* | |
| ICD-10 | Name | ICD-10 | Name |
| F432 | Adjustment disorders | F432 | Adjustment disorders |
| F439 | Reaction to severe stress, unspecified | F439 | Reaction to severe stress, unspecified |
| F999 | Mental disorder, not otherwise specified | F999 | Mental disorder, not otherwise specified |
| F329 | Depressive episode, unspecified | F329 | Depressive episode, unspecified |
| F603 | Emotionally unstable personality disorder | F321 | Moderate depressive episode |
| F419 | Anxiety disorder, unspecified | F419 | Anxiety disorder, unspecified |
| F321 | Moderate depressive episode | F603 | Emotionally unstable personality disorder |
| F609 | Personality disorder, unspecified | F609 | Personality disorder, unspecified |
| F900 | Disturbance of activity and attention | F339 | Recurrent depressive disorder, unspecified |
| F339 | Recurrent depressive disorder, unspecified | F331 | Recurrent depressive disorder, current episode moderate |
| *Fracture or injury* | | | |
| *Offspring* | | *Comparators* | |
| ICD-10 | Name | ICD-10 | Name |
| S934 | Sprain and strain of ankle | S934 | Sprain and strain of ankle |
| S610 | Open wound of finger(s) without damage to nail | S610 | Open wound of finger(s) without damage to nail |
| S600 | Contusion of finger(s) without damage to nail | S600 | Contusion of finger(s) without damage to nail |
| S602 | Contusion of other parts of wrist and hand | S602 | Contusion of other parts of wrist and hand |
| S800 | Contusion of knee | S800 | Contusion of knee |
| S903 | Contusion of other and unspecified parts of foot | S636 | Dislocation of finger |
| S636 | Dislocation of finger | S903 | Contusion of other and unspecified parts of foot |
| S400 | Contusion of shoulder and upper arm | S836 | Sprain and strain of other and unspecified parts of knee |
| S050 | Injury of conjunctiva and corneal abrasion without mention of foreign body | S400 | Contusion of shoulder and upper arm |
| S836 | Sprain and strain of other and unspecified parts of knee | S626 | Fracture of other finger |
| *Intentional or accidental poisoning* | | | |
| *Offspring* | | *Comparator* | |
| ICD-10 | Name | ICD-10 | Name |
| T390 | Poisoning by weak analgesics | T390 | Poisoning by weak analgesics |
| T509 | Poisoning by Other and unspecified drugs, medicaments and biological substances | T509 | Poisoning by Other and unspecified drugs, medicaments and biological substances |
| T659 | Toxic effect of unspecified substance | T659 | Toxic effect of unspecified substance |
| T430 | Poisoning by tricyclic antidepressants | T599 | Toxic effects by Gases, fumes and vapours, unspecified |
| T519 | Poisoning by alcohol, unspecified | T430 | Poisoning by tricyclic antidepressants |
| T399 | Poisoning by Nonopioid analgesic, antipyretic and antirheumatic, unspecified | T399 | Poisoning by Nonopioid analgesic, antipyretic and antirheumatic, unspecified |
| T369 | Poisoning by Systemic antibiotic, unspecified | T369 | Poisoning by Systemic antibiotic, unspecified |
| T599 | Toxic effects by Gases, fumes and vapours, unspecified | T519 | Poisoning by alcohol, unspecified |
| T420 | Toxic effects by Hydantoin derivatives | X601 | Intentional self-poisoning by and exposure to nonopioid analgesics, antipyretics and antirheumatics |
| T439 | Poisoning by Psychotropic drug, unspecified | T398 | Other nonopioid analgesics and antipyretics, not elsewhere classified |
