# Supplementary material for: Adverse health outcomes in offspring of parents with alcohol-related liver disease: Nationwide Danish cohort study
Source: PLoS Med. 2024 Oct 23;21(10):e1004483. doi: 10.1371/journal.pmed.1004483 (PMC11540217; doi:10.1371/journal.pmed.1004483)
Supplement: S4 Table — (DOCX) [file pmed.1004483.s004.docx]

Supplementary Table S4. Incidence rates (IR), incidence rate ratios (IRR) and differences (IRD) for adverse health outcome in offspring (n = 60,804) of patients with alcohol-related liver disease in Denmark 1996 – 2018 and their matched comparators (n = 1,213,356).

| PRIMARY DIAGNOSES ONLY | Events among offspring | IR per 1000 PY among offspring | IRR vs. comparators | IRD vs. comparators per 1000 PY |
| --- | --- | --- | --- | --- |
| Hospital diagnosis |  |  |  |  |
| Psychiatric disease | 46,717 | 34.0 (33.7–34.3) | 1.43 (1.42–1.44) | 10.2 (9.9–10.5) |
| Intentional or accidental poisoning | 6537 | 4.8 (4.6–4.9) | 1.74 (1.69–1.78) | 2.0 (1.9–2.1) |
| Fracture or injury | 195,125 | 142.1 (141.4–142.7) | 1.23 (1.22–1.23) | 26.4 (25.7–27.0) |
| Alcohol-specific | 14,911 | 10.9 (10.7–11.0) | 2.26 (2.20–2.30) | 6.0 (5.9–6.2) |
| Other abuse | 3,453 | 2.5 (2.4–2.6) | 2.29 (2.21–2.37) | 1.4 (1.3–4.5) |
| Death | 1448 | 2.1 (2.0–2.2) | 1.53 (1.45–1.62) | 0.7 (0.6–0.8) |
