# Supplementary material for: Adverse health outcomes in offspring of parents with alcohol-related liver disease: Nationwide Danish cohort study
Source: PLoS Med. 2024 Oct 23;21(10):e1004483. doi: 10.1371/journal.pmed.1004483 (PMC11540217; doi:10.1371/journal.pmed.1004483)

Supplementary Figure S2. Number of offspring of patients with ALD and their matched comparators under observation, by current age.


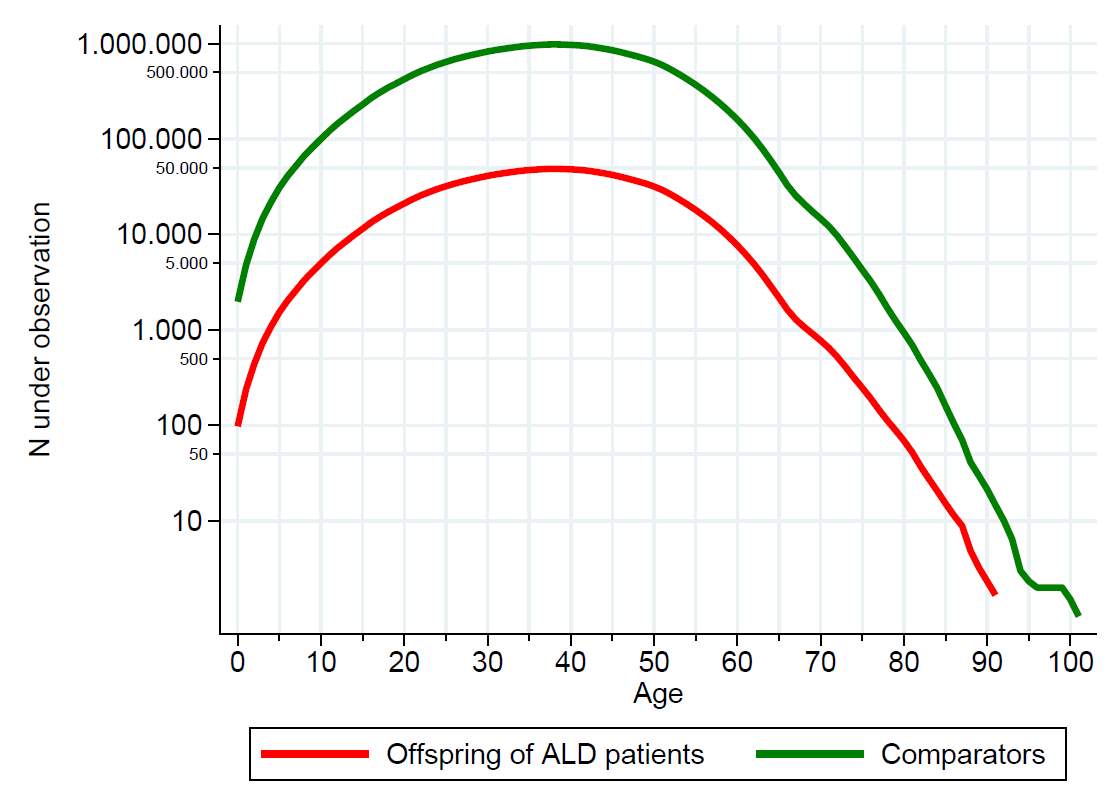

Supplement: S2 Fig — (DOCX) [file pmed.1004483.s006.docx]
