# Supplementary material for: Adverse health outcomes in offspring of parents with alcohol-related liver disease: Nationwide Danish cohort study
Source: PLoS Med. 2024 Oct 23;21(10):e1004483. doi: 10.1371/journal.pmed.1004483 (PMC11540217; doi:10.1371/journal.pmed.1004483)
Supplement: S3 Fig — (DOCX) [file pmed.1004483.s007.docx]

Supplementary Figure S3. Calendar year-specific incidence rate ratios of adverse health outcomes for offspring vs. matched comparators. Calendar year-specific incidence rates are computed separately for offspring and comparators, and the ratio between them (the incidence rate ratio) is shown here with short horizontal lines. The vertical bars illustrate 95% confidence intervals.
