# Supplementary material for: Adverse health outcomes in offspring of parents with alcohol-related liver disease: Nationwide Danish cohort study
Source: PLoS Med. 2024 Oct 23;21(10):e1004483. doi: 10.1371/journal.pmed.1004483 (PMC11540217; doi:10.1371/journal.pmed.1004483)
Supplement: Attachment — Submitted filename: 3.7.24_Comments from editors and reviewers on manuscript.docx [file pmed.1004483.s008.docx]

Comments from editors and reviewers on manuscript

Comment no 1:

Requests from the editors:

1) We share the raised concern, specifically when it comes to the confounders included in your study. If indeed only the socioeconomic position of the parent is taken into account, as one of the reviewer writes, it would be  “... difficult to be confident as to whether the claimed effects are due to ALD, or to (numerous) other confounders (e.g. self/family history of diseases, self alcohol/drug consumption, exercise, etc.) recognized to possibly have effects on the target outcomes (i.e. alcohol-related diagnoses, substance abuse, psychiatric diseases, etc.)” 

Reply:

We agree with the editors that numerous factors may explain the elevated risks in offspring of parents with ALD, and we acknowledge this fact in the discussion, page 15: “Several circumstances could explain why the offspring of parents with alcohol misuse are more vulnerable to adverse health outcomes than the offspring of parents with other chronic diseases: Offspring of parents with alcohol misuse are more likely to encounter psychosocial stressors, such as growing up in a broken family, experiencing domestic violence, being victims of abuse, and suffering material deprivation”.

However, to address this problem we used the case series study design to minimize the effect of unmeasured confounding, as any risk factors present in the offspring before and after the parent’s diagnosis of alcohol-related liver disease are implicitly controlled for. To balance this strength of study design with the complexity of risk factors associated with alcohol use, we have added this comment in the discussion, page 16:

“Our self-controlled analysis was a strong point. In this analysis, every offspring served as his/her own control, minimising confounding by offspring factors that remained unchanged through the study period. Thus, we are confident that a parent’s diagnosis of alcohol-related liver disease is associated with a causal role in the adverse outcomes for the offspring. Even so, the exact nature of this role remains uncertain, and there may be a multitude of other contributing causes. More research is needed to disentangle the many factors that drive the higher rates of adverse health outcomes. Importantly, results from our study highlight the need for preventive interventions and the design of such interventions may not necessarily depend on detailed knowledge of causal factors.”

Overall, our study’s purpose was to describe the risks of adverse health outcomes and resultant need for preventive care—it was not to claim causality. We have now made that clearer in the introduction page 6 by adding “Our aim was to describe these risks and not to address the causes behind them.”

Comment no 2:

2) We also wondered about the use of the diagnosis date (given that the unhealthy alcohol use would almost certainly have preceded this.

Reply: We agree with the editors that hazardous alcohol consumption must have preceded the diagnosis of alcohol-related liver disease. Therefore, we conducted analyses of adverse health outcomes during the 5 years that preceded the ALD diagnosis of the parent until 10 years after the diagnosis, and we have expanded our comment on this analysis in the Results section, page 13:

“For most of the outcomes, the IRR was stable from 5 years before the parent’s ALD diagnosis to 10 years after (Figure 3). On the contrary, there was a sharp increase in the IRR for hospitalization with poisoning in the year after the parent’s diagnosis of alcohol-related liver disease. An increase in the IRR was also seen for hospitalization with psychiatric disease and for death in the year after the parent’s diagnosis of alcohol-related liver disease, but not for the other adverse health outcomes.”

Comment no 3:

3) Please avoid the use “alcohol-related liver disease offspring” in the manuscript and remove ‘nearly lifelong’ from the abstract.

Reply: We have rephrased “alcohol-related liver disease offspring” to “offspring of patients with alcohol-related liver disease” in the method section of the paper, and now this phrase does not appear in the manuscript. The “nearly lifelong” has been rephrased to “long-lasting” in the abstract.

More general request:

Comment no 4:

Data Availability 

PLOS Medicine requires that the de-identified data underlying the specific results in a published article be made available, without restrictions on access, in a public repository or as Supporting Information at the time of article publication, provided it is legal and ethical to do so.

Please see the policy at  

 <http://journals.plos.org/plosmedicine/s/data-availability>  

and FAQs at  

<http://journals.plos.org/plosmedicine/s/data-availability#loc-faqs-for-data-policy>   

 PLOS defines the “minimal data set” to consist of the data set used to reach the conclusions drawn in the manuscript with related metadata and methods, and any additional data required to replicate the reported study findings in their entirety. Authors do not need to submit their entire data set, or the raw data collected during an investigation. Please submit the following data: 

 The Data Availability Statement (DAS) requires revision. For each data source used in your study:  

a) If the data are freely or publicly available, note this and state the location of the data: within the paper, in Supporting Information files, or in a public repository (include the DOI or accession number).
b) If the data are owned by a third party but freely available upon request, please note this and state the owner of the data set and contact information for data requests (web or email address). Note that a study author cannot be the contact person for the data.
c) If the data are not freely available, please describe briefly the ethical, legal, or contractual restriction that prevents you from sharing it. Please also include an appropriate contact (web or email address) for inquiries (again, this cannot be a study author).

In your case, please include an email contact address on how one can make such a request from Statistics Denmark ([www.dst.dk](http://www.dst.dk))

Reply: We have added an email contact address and a more direct link with English information on the data application process by inserting this comment in the Data Availability Statement, page 11: “In accordance with Danish law, we have only been allowed to work with the data on a Trusted Research Environment (in Danish: Forskermaskinen). We are not permitted to download the data, or to share it. However, others can request the same data that we used, and this can be done via an application to Statistics Denmark emailed to [forskningsservice@dst.dk](javascript:mSend('forskningsservice');). More information on the application procedure can be found at (<https://www.dst.dk/en/TilSalg/Forskningsservice/Dataadgang>). The code that we used to conduct the analyses is available upon reasonable request.”


Comment no 5:

Reporting guidance 

Please report your study according to the relevant guidance which can be found here <https://www.equator-network.org/reporting-guidelines/> 

Please ensure that the study is reported according to the STROBE guideline, and include the completed STROBE checklist as Supporting Information.  Please add the following statement, or similar, to the Methods: ""This study is reported as per the Strengthening the Reporting of Observational Studies in Epidemiology (STROBE) guideline (S1 Checklist).

The STROBE guideline can be found here: <http://www.equator-network.org/reporting-guidelines/strobe/>

When completing the checklist, please use section and paragraph numbers, rather than page numbers.
Reply: We have inserted the statement about reporting in the method section just below the Data availability statement, page 11: “This study is reported as per the Strengthening the Reporting of Observational Studies in Epidemiology (STROBE) guideline (Supplementary Table S3).”

Comment no 6:
Prespecified analysis plan/study protocol 


Did your study have a prospective protocol or analysis plan? Please state this (either way) early in the Methods section.  

a) If a prospective analysis plan (from your funding proposal, IRB or other ethics committee submission, study protocol, or other planning document written before analyzing the data) was used in designing the study, please include the relevant prospectively written document with your revised manuscript as a Supporting Information file to be published alongside your study, and cite it in the Methods section. A legend for this file should be included at the end of your manuscript. 


 b) If no such document exists, please make sure that the Methods section transparently describes when analyses were planned, and when/why any data-driven changes to analyses took place.  

 c) In either case, changes in the analysis-- including those made in response to peer review comments-- should be identified as such in the Methods section of the paper, with rationale.

For all observational studies, in the manuscript text, please indicate: (1) the specific hypotheses you intended to test, (2) the analytical methods by which you planned to test them, (3) the analyses you actually performed, and (4) when reported analyses differ from those that were planned, transparent explanations for differences that affect the reliability of the study's results. If a reported analysis was performed based on an interesting but unanticipated pattern in the data, please be clear that the analysis was data-driven. 

Reply:

We did not have prespecified hypotheses of this study and we have now stated that clearly in the introduction page 6. We had a broad analysis plan that formed the basis of the data application to Statistics Denmark, and it was written in Danish. Our aim, as written in this plan, was to compare the health outcomes for offspring of patients with ALD vs. matched comparators and to study the influence of socioeconomic status, but the exact outcomes and the exact methods to make these comparisons were not prespecified in the data application.

We feel that it makes no sense to publish this document in Danish, but we will be happy to share the document with interested readers, which we now state in the method section of the manuscript, page 11: “Our prespecified analysis plan was the data application of the study sent to Statistics Denmark (written in Danish) that can be shared with interested readers upon request to the corresponding author. This plan did not include definition of exact outcomes or exact methods”.

Comment no 7: 
Abstract layout 

Please structure your abstract using the PLOS Medicine headings (Background, Methods and Findings, Conclusions). In the last sentence of the Abstract Methods and Findings section, please describe the main limitation(s) of the study's methodology.

Reply: We have updated the heading of the abstract to match with PLOS medicine headings and inserted this sentence of the main limitation in the Methods and Findings section of the abstract: “Main limitation was inability to include adverse health outcomes not involving hospital contact.”

Comment no 8:

Author summary 

At this stage, we ask that you include a short, non-technical Author Summary of your research to make findings accessible to a wide audience that includes both scientists and non-scientists. The authors summary should consist of 2-3 succinct bullet points under each of the following headings: 

Why Was This Study Done? Authors should reflect on what was known about the topic before the research was published and why the research was needed.
What Did the Researchers Do and Find? Authors should briefly describe the study design that was used and the study’s major findings. Do include the headline numbers from the study, such as the sample size and key findings.  
What Do These Findings Mean? Authors should reflect on the new knowledge generated by the research and the implications for practice, research, policy, or public health. Authors should also consider how the interpretation of the study’s findings may be affected by the study limitations. In the final bullet point of ‘What Do These Findings Mean?’, please describe the main limitations of the study in non-technical language.

The Author Summary should immediately follow the Abstract in your revised manuscript. This text is subject to editorial change and should be distinct from the scientific abstract. Please see our author guidelines for more information: <https://journals.plos.org/plosmedicine/s/revising-your-manuscript#loc-author-summary> 
Reply: An Author summary is now enclosed with the manuscript, immediately following the Abstract.

 Comment no 9:
Introduction layout 

Please address past research and explain the need for and potential importance of your study. Indicate whether your study is novel and how you determined that. If there has been a systematic review of the evidence related to your study (or you have conducted one), please refer to and reference that review and indicate whether it supports the need for your study.

Reply: We now specifically address two relevant systematic reviews in the introduction page 5 and the gaps that we fill with the present study. This is stated here: “Young offspring of parents with alcohol use disorder have higher rates of outcomes associated with stressful life circumstances; poor mental health; and self-harm, including depression, injuries, fractures, heavy alcohol drinking, and drug use, according to a systematic review from 2016 (1,3–5). However, the literature did not cover whether these risks continue throughout adulthood after the child has left home, except for an increased risk of somatic disease in adulthood, reported in more recent studies (6,7).

And here: “According to a recent systematic review, interventions to prevent harm in the offspring of parents with alcohol use disorder often depend on their parent seeking treatment for alcohol use disorder (10,11), but the parent may not do so, and an estimated 40-90% of offspring have not received professional help in relation to their parent’s alcohol misuse (12–14).”

Comment no 10:
Discussion layout 

Please present and organize the Discussion as follows: a short, clear summary of the article's findings; what the study adds to existing research and where and why the results may differ from previous research; strengths and limitations of the study; implications and next steps for research, clinical practice, and/or public policy; one-paragraph conclusion. 
Reply: We have revised the discussion following the PLOS Medicine guidelines.

Comment no 11:
Statistical reporting 

Please quantify the main results with 95% CIs and p values. 

When reporting p values please report as <0.001 and where higher as p=0.002, for example. When reporting 95% CIs please separate upper and lower bounds with commas instead of hyphens as the latter can be confused with reporting of negative values. Please include any important dependent variables that are adjusted for in the analyses. 
Reply: We have inserted p-values in the text and used commas between the lower and upper bounds of 95%CIs.

Comment no 12:
Supplementary materials  

Please note that supplementary materials are not checked and will be posted as supplied by the authors. Therefore, please double check. Please cite your Supporting Information as outlined here:<https://journals.plos.org/plosmedicine/s/supporting-information> - Please note you may use almost any description as the item name of your supporting information as long as it contains an "S" and number. For example, “S1 Appendix” and “S2 Appendix,” “S1 Table” and “S2 Table. Please ensure each supplementary material has a call out (link) from your main manuscript.

Reply: We have read the guidelines on supplementary material and enclosed the supplementary Tables and figures with titles in a single file.

-----------------------------------------------------------

Comments from the reviewers:

Reviewer #1: In this manuscript, the authors examine whether offspring of parents with alcohol-related liver disease had a higher incidence rate of hospital utilization across a series of reasons (alcohol-specific, non-alcohol abuse, injury, etc) using data from the National Denmark cohort registry data. The authors use a sample where offspring are matched to comparators based on age and sex. The results show that offspring of parents with alcohol-specific liver disease had higher incidence rate of contacts with hospitals across a range of reasons, and these associations appear to be stronger among those parents with lower educational attainment. Overall, the findings are interesting, but there are also several concerns and questions regarding the study that should be addressed. These are described below. 

Comment no 13:
(1)     The sample included 60804 offspring. How many of them were siblings from the same nuclear family? Or was one offspring randomly chosen? Additional information on the sampling strategy is needed.
Reply: We included all offspring of patients with ALD, including some that were siblings. Offspring were not included at random. The 60,804 offspring were born to 30,061 different parents who were subsequently diagnosed with ALD. Of those 30,061 parents, 9,063 (30%) had one offspring included in our analysis, 13,270 (44%) had two offspring included, 5,538 (18%) had three offspring included, and 2,190 (7%) had four or more offspring included. We now refer to these results in the Result section page 12. We have rephrased the details of the sampling strategy in the Methods page 7 to make it clearer that we included all offspring born before the ALD diagnosis of the parents, who were diagnosed between 1996 and 2018, and that we excluded offspring born after parental diagnosis of alcohol-related liver disease.

Comment no 14:
(2)     The method states that age- and sex-matched comparators were selected and included in the analyses (20:1 design). It would be helpful to clarify the decision of matching only based on these two demographic factors. It was unclear why the comparators were not matched based on parental socioeconomic status (as indexed by educational attainment).

Reply: We agree with the reviewer that we could have strengthened our study if all 60,804 offspring had been matched to their comparators on parent’s educational attainment. However, this was not possible as when we requested the data from Statistics Denmark, it was not possible to define the ALD-free parent’s educational attainment . Therefore, we opted for the stratified analysis presented in Figure 1. This analysis stratifies the offspring by their parents’ educational attainment, but the comparators are not matched on education. In the revised manuscript, we have added to the discussion page 16 that it was a limitation of our study that we did not match on parental socioeconomic status: “Third, it was a limitation of our study that we did not match comparators and offspring on parental socioeconomic status. However, even offspring of a parent with ALD of higher education had increased risk of hospital contacts due to alcohol-specific, other abuse and psychiatric disease compared to comparators of parents from a broader variety of educational level. Thus, our study findings could not be explained away by confounding by educational level of the ALD parent.”

Comment no 15:
(3)     I believe that the socioeconomic status variable is operationalized as parent's educational attainment. This needs to be clarified throughout the manuscript. Additionally, it states that Table S1 provides a full description of the educational categorization, but this information appears to be missing. This information is crucial, especially in view of results surrounding differences by socioeconomic strata.
Reply: We have now clarified that socioeconomic status is operationalized as parent's educational attainment throughout the paper. We apologize for the missing figure. The description of the educational categorization is now found as Supplemental Figure S1.

Comment no 16:
(4)     Some rationale and justification for using parents' educational attainment as the socioeconomic status. There is social mobility, both inter-generational and intra-generational mobility. Additional clarification would be helpful in understanding this analytic decision.

Reply: We agree that this is important to consider. We have added this paragraph to the Method section, page 7: “We used the educational attainment of the ALD parent as a proxy for socioeconomic status in the offspring. Educational attainment that occurred prior to adulthood in the parent may be less influenced by harmful alcohol consumption during adulthood than are other proxies of socioeconomic position such as employment status and income [9]. It was a limitation that we did not have data on the educational attainment of the offspring. However, an offspring’s final educational attainment may not have been reached at the time of the parent’s ALD diagnosis [10].”

Comment no 17:
(5)     There appears to be some sex differences in the incidence rate ratios. It might be helpful to estimate the IRRs by sex.

Reply: IRRs for all outcomes were estimated by sex and results are found in Figure 1. We have now inserted a comment in the Result section page 12 on the sex difference: “IRRs for adverse health outcomes were higher for male than for female offspring, except for hospital contacts due to fracture or injuries and other abuse than alcohol, for which no difference according to sex was found.”

Comment no 18:
(6)     The self-controlled case series analyses are interesting. For a general readership, a more detailed description of this method would be good.
Reply: We added a more detailed description of the SCCS by inserting these sentences in the Statistical analysis description, page 10: “Briefly, the SCCS method investigates the temporal association between a transient exposure (diagnosis of ALD in the parent) and an event (adverse health outcome in the offspring) (25). Comparisons in the SCCS method are made only within individuals who have experienced an event whereby individuals act as their own control.

Comment no 19:
(7)     How much comorbidity is there among the adverse health outcomes? Some sensitivity analyses censoring all cases with hospital contacts for multiple reasons might provide some robustness check.
Reply: In the revised manuscript we include a sensitivity analysis in which we restricted our analyses to primary diagnosis codes. Upon discharge from the hospital, the physician assigns one primary diagnosis code and up to twenty secondary diagnosis codes to the patient’s hospital contact. Our main analysis did not make a distinction between primary and secondary diagnosis codes, so the reviewer is correct that the hospitalization may not have been *for* something alcohol-related etc., and it could have been for a comorbidity. Our sensitivity analysis is included in the revised manuscript as Supplementary Table S4 and described in the Results section (page 12). Essentially, the incidence rate ratios relative to the comparators are unchanged, so we conclude that the excess hospitalizations are not due to comorbidities.

Comment no 20:
(8)     The authors note that "our findings shown in Figure 1 provide support for a proposed pathway for socioeconomic inequality in health: low socioeconomic position in childhood leads to poor mental health in adolescence; the poor mental health leads to heavy alcohol consumption and smoking in adulthood, and they lead to chronic diseases." While the associations appear to be stronger among those with lower parental SES, I think the results of this study does not necessarily provide direct support for this process-oriented hypothesis. I would revise the text and avoid drawing "causal" conclusions. 
Reply: We agree, we have deleted this paragraph in the discussion.


Reviewer #2: "Adverse health outcomes in offspring of parents with alcohol-related liver disease: nationwide Danish cohort study" analyzes how a parent's diagnosis of alcohol-related liver disease (ALD), affects their offspring in terms of adverse health outcomes within the first year after said diagnosis. National Danish health registry data from 1996-2018 was employed, which provided data for over 60,000 affected offspring. Against age/sex-match comparators, it was found that parents' diagnosis did in fact correlate with an increase risk for adverse health outcomes, particularly when the offspring were of low socioeconomic position.

The findings are relevant towards providing appropriate preventative intervention for vulnerable offspring of ALD parents. However, some issues might be considered:

Comment no 21:
1. The study focuses on "the first year after the parent's ALD diagnosis". It might be commented on whether there might be a (considerable and varying) time lag between the incidence of ALD, and official diagnosis. This would be helpful in considering whether the impact on offspring is due to presence of ALD itself (in which case subsidiary analysis of health outcomes somewhat before official diagnosis might be warranted), or the official diagnosis.

Reply: We agree that there for some patients could be a considerably time lag between incidence of ALD and the hospital diagnosis. We therefore analyzed IRR of adverse health outcomes each year in the five years prior to the parental ALD diagnosis and every year until 10 years after ALD. These results are shown in Figure 3. Our results pointed to a sudden increase in the IRR of poisonings and death during the year of their parent’s diagnosis of ALD, but not obviously in the years leading up to parental ALD diagnosis. In the revised manuscript, we have emphasized this pattern in the Result section, page 13:

“For most of the outcomes, the IRR was stable from 5 years before the parent’s ALD diagnosis to 10 years after (Figure 3). On the contrary, there was a sharp increase in the IRR for hospitalization with poisoning in the year after the parent’s diagnosis of alcohol-related liver disease. An increase in the IRR was also seen for hospitalization with psychiatric disease and for death in the year after the parent’s diagnosis of alcohol-related liver disease, but not for the other adverse health outcomes.”

Comment no 22:
2. Related to the above, analysis of incidence rate (and ratios and differences) against the offspring's previous self (in addition to matched comparators) before the ALD diagnosis, might be appropriate.

Reply: This point raised by the reviewer was exactly the reason why we conducted the self-controlled case series design, which we have expanded the description of in the manuscript. Please see reply to comment number 1 and 18. In this analysis, every offspring served as his/her own control, minimising confounding by offspring factors that remained unchanged through the study period. Thus, we are confident that a parent’s diagnosis of alcohol-related liver disease is associated with a causal role in the adverse outcomes for the offspring. Even so, the exact nature of this role remains uncertain, and there may be a multitude of other contributing causes, and other research is needed to disentangle the many factors that drive the higher rates of adverse health outcomes. Importantly, results from our study highlight the need for preventive interventions and the design of such interventions may not necessarily depend on detailed knowledge of causal factors.

Comment no 23:
3. The significance of the ALD diagnosis might be explained further. Does it generally correlate to a significant change in the patient's ALD status (e.g. diagnosis implies incapacitation or other functional disability, since hospitalization is sometimes used as a synonym, such as in the Interpretation subsection of the Abstract)? This is as one might expect ALD to be a long-term ongoing risk occuring only after a prolonged period of alcohol (ab)use.

Reply: Yes, for most patients diagnosed with ALD in Denmark, the diagnosis correlates with a significant change in the patient’s status. During the period of 1994-2018, most patients were diagnosed in connection with an inpatient admission contrary to an outpatient contact. Most patients with ALD in Denmark have developed cirrhosis at the time of diagnosis (69%) and the majority of these have symptoms due to cirrhosis such as ascites or variceal bleed. We have expanded the description of the clinical stage in the introduction section page 5 by inserting this comment: “Alcohol-related liver disease can progress for years without coming to the attention of healthcare professionals but is often diagnosed when it leads to symptoms of hepatic decompensation of ascites, variceal bleed or liver coma, leading to hospital admission (10,11).

Comment no 24:
4. In the Introduction section, the hypothesis of whether risk to offspring after they have left home was stated as being less-investigated. It might thus be considered to perform stratified analyses on differential risk for offspring still living with parents, and those who do not.

Reply: Unfortunately, we don’t have data on shared household for offspring and their parents with ALD. We have deleted the wording of “after the child has left home” in the Introduction as it is misleading for this study. We have also mentioned the lack of information on shared household as a limitation in the Discussion page 16.

Comment no 25:
5. In the Methods section, it was stated that comparators were matched to offspring on age, sex and birth year. The significance of including both age and birth year might be explained further, since this seems possibly largely redundant.

Reply: We included birthyear as a matching variable because we wanted to limit the influence of differences in the use of hospital care with calendar time (if we had matched on age alone, we could have compared an ALD offspring who was 15 years in 2016 with someone who was 15 years in 1998) . Our study covered the period from 1996 to 2018, that is 22 years in total during which there has been an increase in the number of hospital contacts (<https://www.ncbi.nlm.nih.gov/pmc/articles/PMC7800436/#cit0037>). We have included a comment on this rationale for including birthyear as a matching variable in the manuscript (Method section, description of comparators, page 8): “We chose to match on birthyear in addition to age and sex, since the pattern of hospital care has changed during the study period with an increasing frequency in hospital contacts (10).”

Comment no 26:
6. The full list of confounders might be explicitly specified. From the Data Source section and Table 1, the only confounder appears to be "socioeconomic position of parent (and not the offspring)", which moreover appears to be entirely education-based. Given this, it appears difficult to be confident as to whether the claimed effects are due to ALD, or to (numerous) other confounders (e.g. self/family history of diseases, self alcohol/drug consumption, exercise, etc.) recognized to possibly have effects on the target outcomes (i.e. alcohol-related diagnoses, substance abuse, psychiatric diseases, etc.)

Reply: We have now revised the manuscript to make it clear that the aim of this manuscript was to describe risks and not claim causality for the offspring of ALD parents. Specifically, we have revised the text in the introduction and discussion to reflect this aim. Please see reply to comment number 1 for the specific revisions.

Comment no 27:
7. In the Abstract, the sentence "Offspring of parents with ALD had a nearly lifelong higher rate of health outcomes..." might be rephrased for clarity. In particular, what does "lifelong higher rate" refer to here, since the incidence rate appears to apply only to the period after ALD diagnosis?
Reply: The incidence rates that we refer to covers the period from 15 to 60 years of age. We have rephrased “lifelong” to “long-lasting” in the Abstract.


Comment no 28:
Reviewer #3: This appears to be a revised paper (indicated as PLOSMEDICINE-D-24-01249R1) but I did not review the original submission if this is the case.
Reply: We can assure the reviewer that this is not a revised paper for academic content but we updated an affiliation of one of the co-authors after the initial submission, and this clerical change resulted in a “R1” addition to the manuscript number.

This is a concise paper. It uses a robust administrative data resource (Danish health registry data) to assess the risk of offspring of adults diagnosed with alcohol-related liver disease experiencing adverse health outcomes. It has a longitudinal design, with a case-comparator match design (ratio 20:1). The aim of the paper is to assess whether there is an increased risk for these offspring and when this might be greatest with a view to seizing the opportunity to intervene in order to reduce likelihood of experiencing these adverse outcomes in the future. 

There are a few study design points that are somewhat unexpected for a submission in a publication such as PLOS Medicine. For example, the pre-registration of study design and a priori hypotheses would be expected (although restrictions regarding the sharing of this study data are expected given the sensitive nature of the data and the need for researchers to apply directly to access the data for analysis in secure settings). The research questions are exploratory in nature, although this isn't explicitly stated. It might be advisable to include this detail.

Reply: We agree with the reviewer that our study is exploratory, and we did pre-register the study. To clarify, we have added a comment to the introduction that “we conducted an exploratory study…” (page 6). Also we have added that “Our aim was to describe these risks and not to address the causes behind them and we did not have prespecified hypotheses to test.” (page 6).

Other study strengths include the use of ICD diagnoses for a range of study outcomes (adverse health events, not just alcohol-specific injury or diagnosis). These are listed clearly in the Supplementary document for interested readers.

The self-controlled case series analyses appear to provide the most robust evidence of the 'unique' timing of the one-year interval post-parental diagnosis of alcohol-related liver disease as the highest risk period for offspring (especially those in mid-adulthood). That the risk of adverse outcomes for offspring of those with alcohol-related liver disease compared to other serious health conditions is elevated and persists over the lifespan is an interesting finding. It is also highlighted the compounding effect of low socioeconomic status has for individuals growing up/support by this type of family environment (where a parent has been a heavy/chronic drinker for many years results in liver disease) is not unsurprising but demonstrated empirically via this robust study design. 

I don't have any additional points to improve the quality of the current submission. In my view, it is a relatively simple exploratory paper. The analyses appear to have been well conducted and the take-home message is rightly tentative with respect to potential opportunity/window to intervene with offspring of adults who are diagnosed with alcohol-related liver disease. The fit with PLOS Medicine appears appropriate.

Comment no 29:
Minor comments:
Under 'Results' first paragraph - 1,216,07 match comparators should be 1,216,047 (p.8 of manuscript)
Reply: Thanks for noticing, this is now corrected.
