# Supplementary material for: Adverse health outcomes in offspring of parents with alcohol-related liver disease: Nationwide Danish cohort study
Source: PLoS Med. 2024 Oct 23;21(10):e1004483. doi: 10.1371/journal.pmed.1004483 (PMC11540217; doi:10.1371/journal.pmed.1004483)
Supplement: Attachment — Submitted filename: 12.9.24_R2_Comments from Academic Editor.docx [file pmed.1004483.s009.docx]

Comments from Academic Editor:


This is a large work and ALD is an important topic, likely to be the major cause of liver-related death in the coming decades as viral hepatitis control gets more accelerated.

However, my main issues with this work are as below:

Comment no 1:
1. What is the potential mechanism? It seems a lot are due to socioeconomic and social development factor. For example, the highest rates of problems in offspring occur in the first year from parents' diagnosis. This is likely due to parents being sick and thus diagnosed, absent from home, consuming family financial resources, all of which ultimately lead to resource (both financial and nonfinancial) deprivation which then lead to a variety of problems in offspring. It is hard to infer any biological association from this.

Authors reply: We agree with the editor that a biological association is hard to infer for these offspring risks. Instead, we think parental alcohol use disorder drives poor mental health/stress/depression (as reflected by the increased risk of psychiatric admissions due to these conditions), self-harm (as reflected by the increased risk of admission with poisoning and injury) and substance misuse in the offspring. We have expanded the description of how the outcomes we included are associated with poor mental health and self-harm in the

Introduction, p 6: “Offspring of parents with alcohol use disorder are more likely to experience family dysfunction, material deprivation, domestic violence and childhood abuse (1,3–5). It is therefore not surprising that young offspring of parents with alcohol use disorder have higher rates of outcomes associated with stressful life circumstances; poor mental health; and self-harm, including depression, injuries, fractures, heavy alcohol drinking, and drug use, according to a systematic review from 2016 (1,6–8).”

And also, in the Introduction, p 6: “Parental alcohol-related liver disease may therefore exaggerate mental stress in the offspring which could be observed as an increased risk of hospital admissions with psychiatric disease or intentional poisoning for the offspring at this time point (8,14).”

Comment no 2:
2. What is the clinical relevancy of this study conclusion? How is this going to change practice? I do not believe this study data will change practice. We already know that people with ALD have poor coping skills, poor medical adherence, and often lack social support, all of which make poor parenting and poor family/living situation for children or anyone living with them. It is also already well known that a positive family history of alcoholism is a risk factor for alcohol use disorder in offsprings, so offsprings of people with alcohol use disorder whether they have ALD or not should be offered additional social and school support to cope with their family environment.

(editorial add-on: point 2 should be more acknowledged)

Authors reply: We agree with the editor that this study may not change clinical practice, but it should! What we add to the existing evidence is the issue of timing: We demonstrate that the risk of harm to the offspring is higher immediately after their parent’s ALD diagnosis. This, too, is likely to be the time when both parent and offspring are most receptive to changes in lifestyle and environment. The changes over the offspring’s age are also clinically relevant, e.g., by demonstrating that hospitalizations for poisoning or psychiatric disease take off from an early age indicating that an intervention should be offered from early childhood and not be put off in the hope that ‘the family will sort it out for themselves’. Finally, the lack of improvement over calendar time could—should!—have an indirect clinical impact by demonstrating to policymakers that whatever we have done to date has not worked, thus sparking new policies.

Today, most preventive interventions for the offspring depend on their parent seeking treatment for alcohol use disorder – but they may not do so and this may explain why only 10-60% have received professional help in relation to their parents alcohol misuse (references found in the manuscript). The temporal relationship between a parent’s diagnosis of alcohol-related liver disease and an offspring’s risk of hospitalization due to psychiatric disease or poisoning shows us that there is an optimal time to offer support to the offspring in the clinical setting: It is now! Parent and offspring are likely to be at their peak motivation to change lifestyle and environment when the parent receives a diagnosis of ALD.

We have expanded the description of the clinical relevancy in the Introduction and Discussion section:

We have rephrased this paragraph in the introduction, page 6: “Offspring of parents with alcohol use disorder may not receive the help they need: It is estimated that 40-90% of offspring have not received professional help in relation to their parent’s alcohol misuse (14–16). According to a recent systematic review, this may be due to the circumstance that the offer of professional help to the offspring often depends on their parent seeking treatment for alcohol use disorder, but the parent may not do so (12,13). Therefore, the clinical setting has been suggested as another arena to offer preventive care to the offspring of parents with alcohol use disorder (20). The temporal relationship between a parent’s diagnosis of alcohol-related liver disease and an offspring’s risk of hospitalization due to psychiatric disease or poisoning may show us an opportune time to offer support to the offspring in the clinical setting, hopefully preventing future adverse outcomes.”

And in the Discussion, page 18: “Today, most offspring of parents with alcohol use disorder have not received professional help in relation to their parents drinking (16,17). A parent’s hospital admission for alcohol-related liver disease is a window of opportunity for healthcare professionals to reach out. Few interventions have been tested to improve outcomes in offspring of parents with alcohol misuse (18,19), and the associations we describe have not changed in this millennium (Supplemental Figure S3). We hope that this study will motivate further efforts to provide the help that these offspring obviously need.”

Comment no 3:
Upon re-read the findings a few times: There were various statistical methods of matching and design performed in a large pt cohort. However, it seems to me the main results are higher risk of alcohol related hospital contacts for offsprings of parents with ALD, worse with lower socioeconomic and lower educational levels. It is well known that alcohol use disorder is associated with lower economic status and lower educational level. It is also well known that family history of alcohol use disorder is a risk factor for someone to develop AUD and consequently alcohol related health complications. The etiology of AUD is thought to involve both genetic and environmental factors (Kranzler, Am J Psy 2023, PMID: 37525595).

Authors reply: We agree with the editor that the information that alcohol-related problems run in families is not new, but we do add novel information about socioeconomic status and familial risk of adversity: We demonstrate how the parent’s socioeconomic status affects the risk of adverse outcomes in the offspring. Did you know that also the offspring of highly educated people with ALD had an increased risk of hospitalizations for alcohol-related problems, other abuse, and psychiatric disease (not for poisoning)? We did not. These observations might help us alleviate the stigma that alcohol-related problems are confined to families of lower socioeconomic status.

We have changed the order of the outcomes throughout the paper and in the abstract to increase the attention to outcomes associated with poor mental health and self-harm in the offspring, that is admission with psychiatric disease, poisoning and injury. We have expanded the Introduction and Discussion as well with descriptions of these links, see reply to Comment no 1. We have also described the most common diagnoses of hospital contacts for poisoning and psychiatric disease in the result section to clarify that hospital admissions with poisoning are due to self-harm with medications, and hospital contacts with psychiatric disease are due to stress-related disorders as well as depression and anxiety.

This paragraph is inserted in the Results section, p 13: “Hospital contacts due to poisoning were most frequently due to poisoning with medications that are intentional in their nature whereas hospital contacts with psychiatric disease were most frequently due to stress, depression or anxiety (Supplementary Table S2).”

Comment no 4:
In smaller detail, in response to editor comment #2 in Revision 2, the authors expanded analyses to include health outcomes 5 years before ALD diagnosis and 10 years after and found the results to be stable. This would suggest that that it was not that index ALD diagnosis analyzed in this study that was the issue but the likely lifelong exposure to alcohol use disorder and associated family dysfunction and other genetic factors that were the problems.

Authors reply: We agree that the incidence rate ratios were nearly stable around the parent’s diagnosis of ALD for most of the outcomes (as shown in Figure 3) – but not for poisoning and psychiatric disease. However, our self-controlled analyses regarding the risk of a first adverse health outcome according to offspring age (shown in Table 3), confirmed that offspring were generally more likely to experience their first adverse health outcome during the year following their parent’s diagnosis of alcohol-related liver disease than they were at another time.


Comments from Reviewers:

Reviewer #1: My comments have been adequately addressed. I think this manuscript will make a fine contribution.


Reviewer #2: We thank the authors for addressing our previous comments, and acknowledge our misconception for the self-controlled case series design originally raised in Point 2. Concerns on sociodemographic factors were covered in the responses to Reviewer #1.


Reviewer #3:  I am happy the authors have addressed my requests for minor revisions.
 
The paper is suitable for publication now, in my opinion.
